# Genome-wide transcriptomic analysis reveals correlation between higher WRKY61 expression and reduced symptom severity in Turnip crinkle virus infected *Arabidopsis thaliana*

**DOI:** 10.1038/srep24604

**Published:** 2016-04-18

**Authors:** Ruimin Gao, Peng Liu, Yuhan Yong, Sek-Man Wong

**Affiliations:** 1 Department of Biological Sciences, National University of Singapore, Singapore; 2 Temasek Life Sciences Laboratory, Singapore; 3 National University of Singapore Suzhou Research Institute, Suzhou Industrial Park, Jiangsu, China

## Abstract

Turnip crinkle virus (TCV) is a *carmovirus* that infects many *Arabidopsis* ecotypes. Most studies mainly focused on discovery of resistance genes against TCV infection, and there is no Next Generation Sequencing based comparative genome wide transcriptome analysis reported. In this study, RNA-seq based transcriptome analysis revealed that 238 (155 up-regulated and 83 down-regulated) significant differentially expressed genes with at least 15-fold change were determined. Fifteen genes (including upregulated, unchanged and downregulated) were selected for RNA-seq data validation using quantitative real-time PCR, which showed consistencies between these two sets of data. GO enrichment analysis showed that numerous terms such as stress, immunity, defence and chemical stimulus were affected in TCV-infected plants. One putative plant defence related gene named *WRKY61* was selected for further investigation. It showed that WRKY61 overexpression plants displayed reduced symptoms and less virus accumulation, as compared to wild type (WT) and WRKY61 deficient lines, suggesting that higher *WRKY61* expression level reduced TCV viral accumulation. In conclusion, our transcriptome analysis showed that global gene expression was detected in TCV-infected *Arabidopsis thaliana*. *WRKY61* gene was shown to be negatively correlated with TCV infection and viral symptoms, which may be connected to plant immunity pathways.

Plants have to defend themselves against pathogens in order to survive over time. Some defenses are achieved through producing antimicrobial compounds^1^, and others are by recognition of pathogens^2^,^3^. The key players in the recognition process includes product of a dominant or semi-dominant resistance (R) gene present in the plant or the corresponding dominant *avirulence* (*Avr*) factor encoded by or derived from the pathogens^4^. Recognition of the *Avr* factor by the host plant initiates one or more signal transduction pathways that activate various plant defenses and thus compromise the ability of the pathogens to colonize the plants^5^. Understanding various gene regulation network will facilitate researchers to know the underlying molecular mechanisms of virus-plant host interactions.

*Turnip crinkle virus* (TCV) is a plant virus belonging to *Tombusviridae* Family *Carmoviruses*. It infects many *Arabidopsis* ecotypes^6^. TCV induces a hypersensitive reaction (HR) at 2 to 3 days post inoculation (dpi) in *Arabidopsis* ecotype Dijon (Di-0 and Di-17)^7^,^8^,^9^. A dominant gene, HRT, which confers an HR to TCV, has been identified and mapped in the Di-17 line of Dijon to a region 2 cM telomeric of the DFR marker on chromosome 5^10^. In addition, HRT gene was also identified to trigger defence gene expression and SA accumulation^6^,^11^,^12^,^13^,^14^. A genetic screening for mutants with compromised HRT-mediated resistance identified crt1 (compromised for recognition of TCV) and CRT1 was subsequently shown to interact with HRT and ten other R proteins representing three different structural classes^15^,^16^,^17^. For host plant, basal defences to TCV often involves RNA silencing, which is induced upon the formation of double stranded RNA which is processed further to small 20–30 nucleotide dsRNA^18^,^19^,^20^. A potential RNA silencing suppressor, TCV coat protein (CP) can activate R protein HRT^21^,^22^, but not through its silencing function of CP^23^. However, it is indicated that double-stranded RNA-binding protein (DRB) 4, which is the component of the host RNA silencing pathway, participate in HRT-mediated defense against TCV^24^. It has also been found that TCV-CP can directly interact with a NAC transcription activator-like protein named TIP (TCV-interacting protein) and inhibit the nuclear localization of TIP^22^,^25^.

The interaction between plants and viral pathogens reflects a sophisticated coevolution of recognition, defense, and counter-defense mechanisms. Although plant viruses are among the least genetically complex pathogens, they use a variety of strategies to suppress or bypass host defense and infect susceptible hosts. In plants, these strategies involve enhancing infection by manipulating host resources, such as the formation of replication complexes^26^, enlargement of the plasmodesmata size-exclusion limit^27^,^28^, evolution of viral suppressors of RNA silencing to counteract antiviral silencing^29^, interference with regulation of the plant cell cycle^30^,^31^, and using host components for its own replication^32^. In turn, plants have evolved intricate mechanisms to fight viral infection, such as pathways mediated by gene silencing, hormone-mediated signaling pathways, and regulation of metabolism^19^,^20^,^33^,^34^. WRKY transcription factors comprise one of the largest families of regulatory proteins in plants and a total of WRKY transcription factors comprise one of the largest 72 WRKY-encoding genes were identified in *Arabidopsis thaliana*^35^. The conserved WRKYGQK sequence of WRKY protein is directly involved in DNA binding^36^, and the zinc finger motif is also required^37^. Most of the reported WRKY proteins bind to the W-box element (C/T)TGAC(C/T) in the promoter region of the target genes^38^. WRKY8 has been shown to be involved in the defense response against TMV-cg and may mediate the crosstalk between ABA and ethylene signaling during the TMV-cg-*Arabidopsis* interaction^39^. A report has shown that the WRKY45-2-WRKY13-WRKY42 transcriptional regulatory cascade is required for rice resistance to fungal pathogen^40^.

Genome wide analysis has becoming a promising tool to discover global gene change profiles in plant pathology discoveries. For example, an RNA-sequencing based transcriptome analysis of compatible interaction of tomato (*Solanum lycopersicum*) and *Verticillium dahlia* facilitates researchers to identify key susceptible genes and understand the mechanism of tomato susceptible response to *V. dahlia*^41^. Analysis of the tomato leaf transcriptome during successive hemibiotrophic stages of a compatible interaction with the oomycete pathogen *Phytophthora infestans* will provide a broad view of the hemibiotrophic infection process from the plant perspective^42^. Transcriptome analysis of the fungal pathogen *Fusarium oxysporum* f. sp. *cubense* causing banana vascular wilt disease will assist researchers to identify pathogenicity mechanism involved in the disease development^43^. Genome-wide transcriptome analysis of the plant pathogen *Xanthomonas* has also identified a group of small RNAs with putative virulence functions^44^. In addition, a genome-wide alternative splicing (AS) analysis provides new insights into AS landscapes conserved among monocots and dicots and uncovered AS events in plant defense-related genes during plant-microbe interaction^45^. Among the identified genes, over 100 immune-related genes encoding receptor-like kinases, NB-LRR resistance proteins, transcription factors, RNA silencing, and splicing-associated proteins were identified^45^. Furthermore, a mixed transcriptome analysis is also very useful for the simultaneous elucidation of the tactics of host plant defense and pathogen attack during rice blast fungus (*Magnaporthe oryzae*) interaction^46^. Although some microarray data were used to carry out comparative analysis in *Arabidopsis* transcriptome with plant viruses infection^47^, there is no Next Generation Sequencing (NGS) based high throughput transcriptome analysis for TCV infection study. Thus, Illumina high-throughput mRNA sequencing (RNA-seq) technology was used in this study to identify globally differentially expressed genes (DEGs) between TCV-infected *Arabidopsis* compared to mock plants.

A NGS based genome wide transcriptome analysis will not only provide us with the global gene change profiles containing a list of the up- and down-regulated gene candidates, it also displays gene ontology (GO) enrichment analysis and related gene network that are involved in TCV infection. This study will provide researchers with more information for better understanding of the mechanism of virus-host interaction.

## Results

### Raw sequencing data and mapping of RNA-seq reads to *Arabidopsis* genome

To reveal the TCV infection-associated transcriptional responses in *Arabidopsis*, both mock and TCV-infected samples were subjected to total RNA extraction and RNA-seq analysis. The single lane of Illumina HiSeq2000 high-throughput sequencing generated 155.26 and 170.38 million paired-end reads (2 × 101 bp) for mock and virus-inoculated samples, respectively. Using FastQC program to evaluate the reads quality, it showed decent quality data. Some representative results were shown in Supplementary Document 1. For the obtained 101bp reads, as observed from the per-base quality graph, all of them were present in the green color section, suggesting the good base pair quality obtained in each read (Supplementary Document 1). To guarantee better results for the following mapping and assembly, the raw reads were further trimmed and filtered, a total of 9.9% of reads were discarded, leaving over 310 million reads for downstream analysis (2 × 91 bp). The obtained reads were mapped to the *Arabidopsis* genome using TopHat and the alignment summary were shown that around 97.5% of input reads from mock plants were mapped to *Arabidopsis* genome, and around 89.5% reads were mapped in TCV-infected sample reads (Supplementary Document 1). The relatively lower mapping rate in TCV samples was due to some viral reads were also present in the TCV-infected samples.

### Identification of differentially expressed genes between mock and TCV-infected *Arabidopsis*

**Figure 1:**
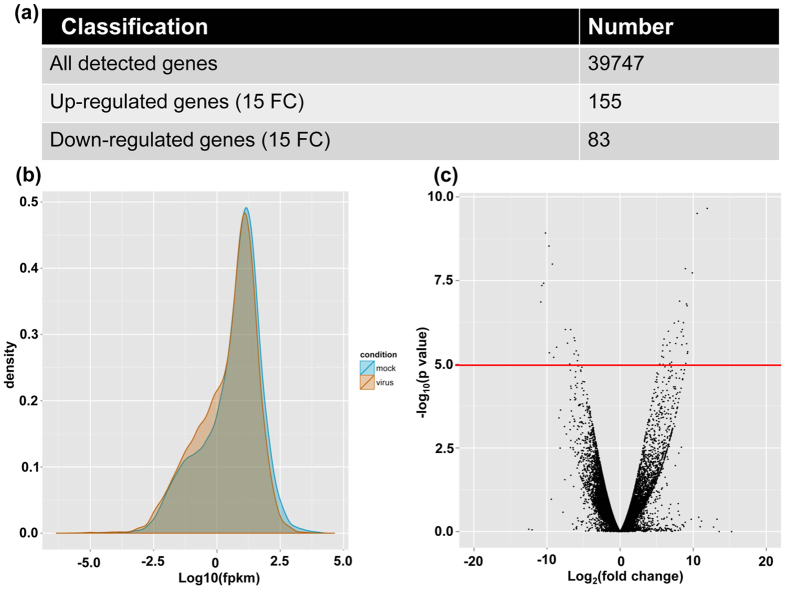
Transcriptome analysis of RNA-seq data of mock and *Turnip crinkle virus* (TCV)-infected *Arabidopsis thaliana*. (**a**) Numbers of all the detected genes and differentially expressed genes (DEG) with at least 15-fold change (FC) in mock and TCV-infected *Arabidopsis* plants. (**b**) Plot for the distribution and comparison of Fragments per kilobase of transcript per million fragments mapped (FPKM) values for genes expressed in the mock and TCV-infected plants. (**c**) Volcano plot to inspect DEGs under different conditions between mock and TCV-infected plants. For instance, the genes which are located above the red line represent the DEGs under conditions of log2 (FC) >4 and p < 0.00005.

The mapping output generated by TopHat was processed by Cufflinks toolkits for transcript assembly and differential gene expression analysis. In general, a total of 39,747 expressed genes were detected from the mock and TCV-infected samples (Fig. 1a, Supplementary Table S1). In addition to the well-known 27,677 protein coding genes in *Arabidopsis*, this 39,747 discovered gene transcripts also included non-coding RNA genes, such as miRNA genes, siRNA genes and long non-coding RNA genes. In order to narrow down the DEG list, the genes with at least 15-fold change (FC) were selected for further investigation. There were 238 genes in total and the numbers of up- and down-regulated genes in TCV-infected samples were 155 and 83, respectively (Fig. 1 and Supplementary Table S2). Many functional classes of genes, such as ATNADP-ME1 (NADP-malic enzyme 1), ALPHA-DOX1 (peroxidase superfamily protein), ALD1 (AGD2-like defense response protein 1), stress response protein (stress responsive A/B barrel domain) as well as a few WRKY family proteins, were found to be significantly affected in TCV-infected plants compared to that of the mock control plants (Supplementary Table S2).

### Gene ontology (GO) enrichment analysis of differentially expressed genes

**Figure 2:**
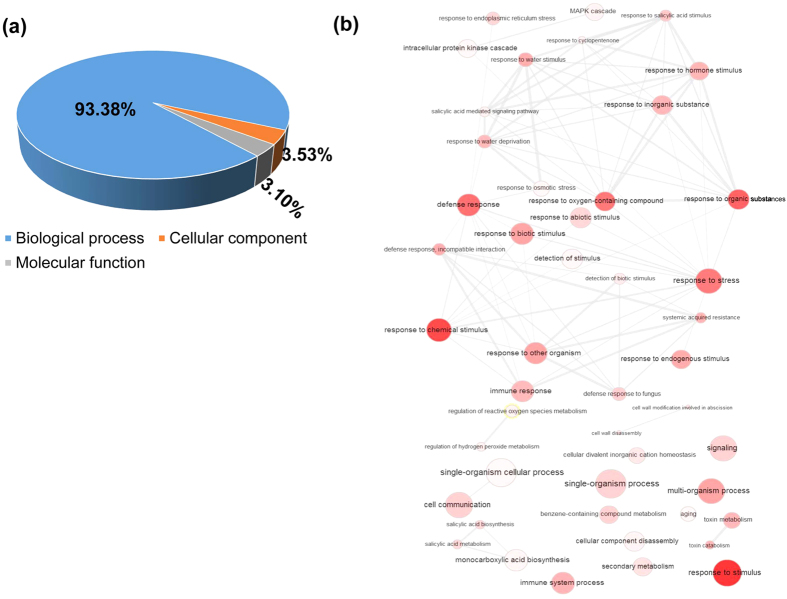
Gene ontology (GO) enrichment analysis of DEGs which were significantly affected (up- and down- regulated >15 FC) by TCV infection. (**a**) Fraction distribution of DEGs related GO terms based on molecular function, biological process, and cellular component. (**b**) Interactive graph of biological process of 238 DEGs which are involved in the TCV infection. The size and red colour density of the circles are proportional to the number of genes associated with the specific term. The interactive network were summarized and plotted following published REVIGO protocol (http://revigo.irb.hr).

The differentially expressed 238 genes with at least 15-FC were used to carry out GO enrichment analysis. There are approximately 1389 GO terms were assigned, and the fraction percentages for biological process (1297), cellular component (49) and molecular function (43) were 93.38%, 3.53% and 3.10% respectively (Fig. 2a). Of 1297 terms in the biological process, there were 207 terms, such as immune system process (19), response to stress (50), defense response (47), immune response (17), innate immune response (17), response to biotic stimulus (27), systemic acquired resistance (14), regulation of immune system process and regulation of innate immune response (16) and regulation of immune response (8) were directly related to plant immunity after TCV infection. The full list of the GO terms for these three fractions (biological process, cellular component and molecular function) was shown in Supplementary Table S3. In order to display the correlations of the interesting biological process GO terms, their interactive graph was shown in Fig. 2b. It displayed some key GO terms such as stress, immune response, defence response and chemical stimulus which played critical roles in the network regulation of differentially expressed GO terms, indicating that intricate regulation relationship could be present in TCV-host interaction and plant virus infection may also simultaneously influence other abiotic stress pathways in plants.

### Validation of RNA-seq data by quantitative real time PCR (qRT-PCR)

**Figure 3:**
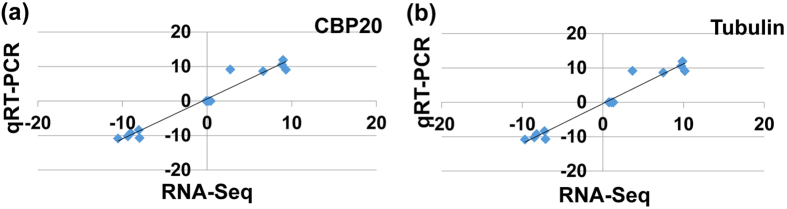
Validation of RNA-seq data using qRT-PCR. Two internal controls (**a**) *CBP20* and (**b**) *tubulin* genes were used to validate the RNA-Seq data. Fold changes of gene expression detected by RNA-seq were plotted against the data of qRT-PCR. The reference line indicates the linear relationship between the results of RNA-seq and qRT-PCR analyses.

In order to validate the data from RNA-seq analysis, among all the 238 DEGs, five genes were randomly selected from the top 20 most significantly upregulated or downregulated genes, respectively (Supplementary Table S2). Five genes were randomly selected from the non-significant changed gene category (Supplementary Table S1). In total, fifteen genes (five upregulated, five with no significant changes and five down-regulated) were randomly selected for expression analysis by qRT-PCR. The expression data for these selected genes between RNA-seq and qRT-PCR data were shown in Supplementary Table S4. In general, there is a strong correlation between these two sets of data, which was shown by a linear relationship for the gene expression (both CBP20 and tubulin were used as internal controls) (Fig. 3), suggesting the qRT-PCR and RNA-seq data exhibited decent agreement in all of the randomly selected upregulated, normal expression and downregulated genes.

### Validation of *WRKY61* gene in TCV-infected plants over a time course of 16 days

**Figure 4:**
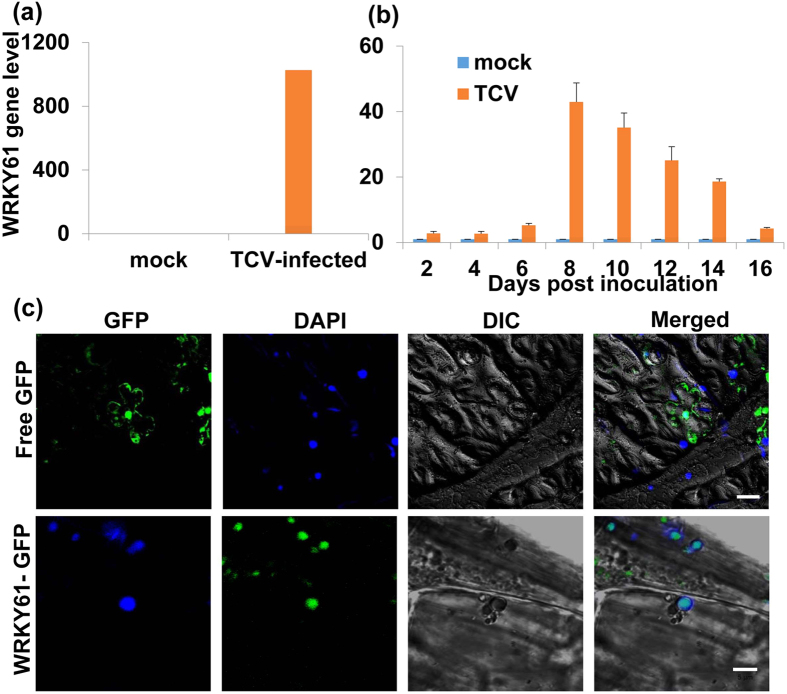
Relative gene expression level of WRKY61 in TCV-infected *Arabidopsis* and nuclear localization of WRKY61-GFP fusion protein in *Nicotiana benthamiana* leaves. (**a**) The qRT-PCR was used to validate *WRKY61* gene expression. (**b**) Expression of *WRKY61* gene transcript after TCV infection as determined by qRT-PCR. The gene transcript level of *WRKY61* was shown at 2, 4, 6, 8, 10, 12, 14 and 16 days post inoculation (dpi). Relative gene transcript levels (CBP20 and Tubulin as internal controls) were analyzed using the 2^−∆∆C^_T_ method. The values of *WRKY61* in TCV-infected plants were calculated by subtracting the values from mock controls which were all set to 1 for standardization. Means of three independent biological repeats were shown with standard deviations. (**c**) DAPI-stained nuclei (blue-color foci) were superimposed onto the differential interference contrast (DIC) image to form a merged image. *N. benthamiana* leaves were infiltrated with free GFP and the fluorescent signal was present in the entire cell including nucleus; WRKY61-GFP fusion proteins were only detected in the nucleus. Free GFP represents agro-infiltration with vector lacking of inserted gene. Bar = 5 μm.

Among 238 most significantly expressed genes (>15 FC) after TCV infection, a transcription factor *WRKY61* (AT1G18860) was selected for further investigation. The reason for choosing *WRKY61* is that it is a transcription factor that displayed significant upregulation and WRKY gene families are reported to be involved in plant immunity^48^. Using qRT-PCR, the expression level of *WRKY61* was validated to be consistent with the transcriptome RNA-seq analysis data (FC: 414.42). (Fig. 4a, Supplementary Table S2). In order to further understand the *WRKY61* gene expression level among TCV infection process, a time course of 16 dpi experiments were selected for investigation. The results showed that the gene transcripts of WRKY61 were increased after TCV infection and reached its highest expression level at 8 dpi and declined after that (Fig. 4b). It suggests that the gene expression of *WRKY61* responded positively after TCV infection.

### Localization of WRKY61-GFP fusion protein

In order to further understand this putative transcription factor WRKY61, its subcellular localization was determined using *Agrobacterium*-mediated transient expression in *Nicotiana benthamiana* plants through leaf infiltration. Firstly, *WRKY61* gene was inserted to a binary vector named 35SpGreen-GFP to form a construct 35SpGreen + WRKY61-GFP which was transformed into agrobacterium strain GV3101 (Agro-35SpGreen + WRKY61-GFP). The required primers were listed in Supplementary Table S5. The transient expression results showed that green florescent signal was detected in the entire cell including nucleus when the leaf cells were infiltrated with a free GFP (Agro-35SpGreen) (Fig. 4c, upper row). When the *N. benthamiana* leaf cells were infiltrated with Agro-35SpGreen + WRKY61-GFP, the GFP signal from the fusion protein was only detected in the nucleus (Fig. 4c, bottom row), demonstrating that WRKY61-GFP fusion protein was localized only in the nucleus.

### Comparison of virus resistance among WT, WRKY61-deficient and WRKY61 overexpression plants

**Figure 5:**
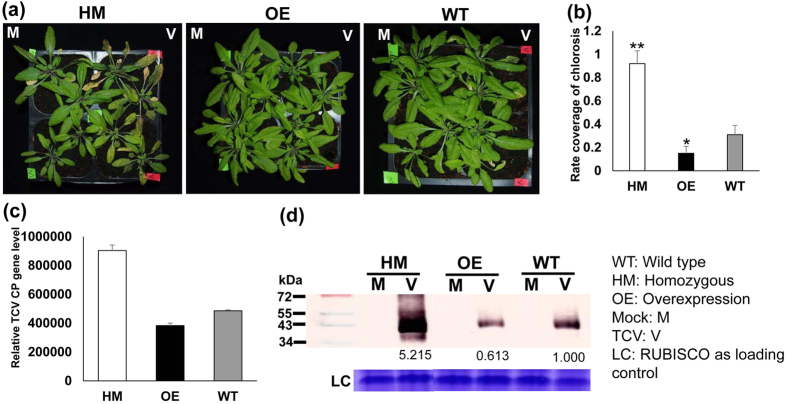
WRKY61 is positively correlated with virus resistance after TCV infection. (**a**) Comparison of TCV symptoms on *Arabidopsis* with different backgrounds [WT, *WRKY61* deficient (homozygous HM), and overexpression(OE)] at 10 days post inoculation. TCV symptoms were not observed in all the mock control plants. Only TCV infected leaf displayed chlorosis. (**b**) Statistical analysis of the percentages coverage of chlorosis in each leaf of TCV infected HM, OE and WT plants. Six leaves from each plants (a total of 8 plants from each treatment) were evaluated. The student *t* test was used to analyze the significant differences between WT and WRKY61-deficient HM lines, as well as WT and WRKY61 OE lines (*p < 0.05, **p < 0.01). Detection of TCV-CP in newly emerged leaves of three types of plants WT, WRKY61 deficient (HM), and overexpression (OE) line at 10 dpi from both RNA (**c**) and protein (**d**) levels. The transcript level of *TCV-CP* gene was analyzed using qRT-PCR. Relative gene transcript levels (*CBP20* and *Tubulin* as internal controls) were analyzed using the 2^−∆C^_T_ methods. Means of three independent biological repeats were shown by standard deviations. Detection of TCV-CP protein in the WT, WRKY61 deficient (HM) and OE lines were analyzed using western blot. The protein band intensity was quantified using ImageJ software. Ribosome was used as loading control.

To verify if WRKY61 is involved in conferring virus resistance, TCV inoculation was carried out in WT, WRKY61 deficient (homozygous: HM), and WRKY61 overexpression (OE) plants. The selected WRKY61 HM deficient line was screened and verified from TAIR seed stock T-DNA insertion mutant (Stock CS459288, individual line CS360187). The primers used for screening HM mutant were listed in Supplementary Table S5. The confirmed WRKY61-deficient HM lines were further analyzed by qRT-PCR which showed that no *WRKY61* gene transcripts can be detected. For the WRKY61 overexpression lines, 2 × 35S-WRKY61-6HA construct was successfully transformed into *Arabidopsis* plants. The overexpression of *WRKY61* gene transcript was confirmed using qRT-PCR and the fusion protein of WRKY61-6HA was also detected in 4 lines of WRKY61-6HA transgenic plants tested (Supplementary Fig. S1), indicating successful transformation of WRKY61-6HA to *Arabidopsis* plants. For virus resistance testing, WRKY61 HM deficient lines, WT and WRKY61 OE lines (3 lines, L2, L3 and L4 were selected for testing) were inoculated with equal amount of TCV *in vitro* transcripts (400 ng) onto each plant. Compared to WT and WRKY61-deficient mutants, all three WRKY61 OE lines showed similar viral tolerance upon TCV infection. Thus, data from L3 plants were shown as a representative in this study. The results showed that the plants of WRKY61 (HM) deficient mutants displayed the most severe symptoms, compared to that of WT control which appeared to be similar to the symptoms of TCV-infected WRKY61 OE plants (Fig. 5a). The viral symptom comparisons were further demonstrated by the percentages of chlorotic lesions covered in the leaves of TCV-infected plants, which showed TCV-infected WRKY61 (HM) lines contain the highest percentage of chlorosis coverage (Fig. 5b), followed by WRKY61 OE and WT plant control. In addition to the direct symptom observation, the *TCV-CP* gene transcripts were further assessed by qRT-PCR, which also showed the most abundance *TCV-CP* gene transcripts were detected in WRKY61 HM deficient mutants (Fig. 5c). Furthermore, these virus tolerance results were also confirmed with Western blot analysis, showing the relative amount of CP expression (Fig. 5d). Although the symptom of WRKY61 OE plants appeared to be similar to that of WT, the amount of *CP* gene transcript and CP expression is less than that of the WT.

## Discussion

In this study, we have shown that different WRKY61 expression level is closely related to the virus tolerance after TCV infection. However, among the three WRKY61 OE lines, there is no great differences with regard to the tolerance to virus resistance, indicating that there may be a threshold for the amount of *WRKY61* gene expression to combat virus resistance. As a subgroup IIb in the *Arabidopsis WRKY* gene family, *WRKY61* is a potential candidate for us to study TCV-host interaction. In *Arabidopsis* WRKY family, with a closer relationship to subgroup IIb is the well-studied WRKY family protein of subgroup IIa, which mainly contains WRKY18, WRKY40 and WRKY60. These three *Arabidopsis* WRKY proteins are reported to be involved in stress responses, which include resistance against bacteria *Pseudomonas syringa*e and fungus *Botrytis cinerea*^49^,^50^. AtWRKY18 is a salicylic acid-induced gene that positively regulates system acquired resistance (SAR) and modulates *pathogenesis-related (PR)* gene expression^51^,^52^; AtWRKY18 overexpression increases resistance to *P. syringae*^53^. AtWRKY40 and AtWRKY60 proteins antagonize AtWRKY18 during *P. syringae* infection. The gain or loss of gene function in single, double or triple combination mutants resulted in increased susceptibility to *Botrytis cinerea*^49^. In addition to *Arabidopsis*, some rice (*Oryza sativa*), barley (*Hordeum vulgare*) and *Brassica napus* WRKY members from group IIa are also involved in the response to fungal and bacterial pathogens, showing that *OsWRKY62* and *OsWRKY76* are upregulated in *Magnaporthe grisea* infected-leaves and downregulated in *Xanthomonas oryzae*-inoculated leaves^54^. HvWRKY1 and HvWRKY2 play an important role in response to *Blumeria graminis* infection^55^, and BnWRKY18 and BnWRKY40 play a role in the response to *Sclerotinia sclerotiorum* and *Alternaria brassicae* infections^56^. Furthermore, a promising role of WRKY transcription factors discovered in plant immunity was that they could be targeted by pathogen effectors which were detected by a plant immune receptor^57^, this integrated WRKY domain of *Arabidopsis* RRS1-R NB-LRR protein functions as a decoy to detect pathogen interference^57^,^58^. In our study, we have shown that the upregulation of *WRKY61* ameliorated viral symptoms after TCV infection, similar to other plant species, it is possible that WRKY61 may also paly similar roles in regulating SAR and modulates PR gene expression^52^,^53^. It will be interesting to discover novel roles of WRKY transcription factor in plant virus infection. This study may provide valuable clues for further investigation of TCV-host interaction.

## Conclusion

In summary, by using genome wide comparative transcriptome analysis, a global gene expression change profiles were detected between mock and TCV-infected *Arabidopsis* plants. Among them, 238 DEGs with at least 15-FC were determined. GO enrichment analysis showed that the differentially expressed GO terms are involved in different pathways including stress, immune response, defence response and chemical stimulus, indicating plant virus infection is also closely related to the other abiotic stress pathways in plants. One of the most significant DEGs named *WRKY61* was shown to be upregulated after TCV infection. Similar to other plant species, it is possible that WRKY61 may also paly roles in regulating SAR and modulates PR gene expression. Our genome-wide transcriptome analysis provides large set of database information which may provide novel clues for further investigation the molecular mechanisms underlying TCV infection in *Arabidopsis*. It has also uncovered many new putative defence response genes which may play essential roles in TCV infection.

## Methods

### Plant materials, plasmid construction and generation of transgenic *Arabidopsis*

*Arabidopsis* plants were grown under long-day conditions (16 h light/8 h dark cycles) at 23 °C, humidity 70%. The *WRKY61* gene was PCR amplified with appropriate primers listed in Supplementary Table S5. The PCR product was ligated to OE 2 × 35S–6HA vector or 35S pGreen-GFP vector to generate OE 2 × 35S-WRKY61-6HA vector and 35S pGreen-WRKY61-GFP, respectively. The generated constructs was transformed individually into *Arabidopsis* ecotype Col-0 using the floral dip method^59^.

### Library construction and high-throughput RNA sequencing

*Arabidopsis* leaves were collected (three individual biological repeats) at 8 dpi for RNA extraction with TRIZOL reagents (Invitrogen), following the manufacturer’s instructions. Total RNA contents were measured using the NanoDrop 2000C (Thermo Scientific) and the quality of RNA samples was assessed by agarose gel electrophoresis. Before library construction, the integrity of RNA samples was confirmed using Agilent Bioanalyzer 2100 RNA Nano chip (Agilent Technologies). Following same amount of total RNA from each biological repeat were pooled together for one library construction. The poly(A) RNA from 500 ng of total RNA was enriched with oligo dT beads and then fragmented to convert to double stranded cDNA followed by library construction. One ul of final libraries was loaded on a Bioanalyzer 2100 DNA High Sensitivity chip (Agilent Technologies) to check for size. Finally, each library (from mock and TCV-infected plants) was loaded in one lane separately and sequenced on an Illumina HiSeq2000 platform (Illumina) using BGI services.

### Differential gene expression analysis

Using *Arabidopsis* genome as references, the differential gene expression analysis was based on the published protocols^60^. Briefly, raw sequencing data were firstly evaluated with FastQC program. Then the reads were 5′ trimmed on quality score (Q > 30), adapter sequences removed and short reads dropped using NGS QC toolkit (v2.2.3)^61^. All filtered and properly paired reads were then mapped to the *Arabidopsis* genome using TopHat (V2.0.10). The fragment alignments generated by TopHat were used as input files for Cufflink (V2.2.1) and further analyzed through the recommended pipeline to detect the differentially expressed genes between mock and TCV-infected plant samples. The detailed steps and parameters used for the analysis were listed in Supplementary Document 1.

### GO enrichment analyses

All the genes identified with significant differential expression (p < 0.0005) and fold-change >15 in this study were used as input to carry out GO enrichment analysis. The function of batch gene analysis was used from Gene Ontology Enrichment Analysis Software Toolkit (GOEAST) (http://omicslab.genetics.ac.cn/GOEAST/php/batch_genes.php). GOEAST uses defaulted hypergeometric test to calculate p-values or FDRs and it provides FDR value with multi-test adjustment. The enriched GO terms were summarized and plotted following the published REVIGO protocol (http://revigo.irb.hr)^62^. The fraction ratios of cellular component, biological process and molecular functions were calculated based on the number of GO terms.

### *In vitro* transcription of TCV transcripts using Pt1d1 plants and virus inoculation

The full-length cDNA clone pt1d1^63^ was linearized with *XbaI*, linearizing the TCV full-length cDNA clone with a T7 RNA polymerase promoter immediately adjacent to the 5′-terminus. The fragments were purified and transcribed *in vitro* using T7 RNA polymerase (Ambion, mMESSAGE mMACHINE) under the reaction conditions recommended by the manufacturer. *In vitro* transcribed RNA was verified for integrity by gel electrophoresis. Then the transcribed RNA (400 ng) was mixed with equal volume of 2 × GKP buffer (50 mM glycine, 30 mM K_2_HPO_4_, pH 9.2, 1% bentonite and 1% celite). Three fully expanded true leaves from healthy *Arabidopsis* plants were inoculated. In addition, plants were also inoculated with 1 × GKP buffer as mock (negative control). Immediately after inoculation, plants were rinsed with sterile water and placed under low light. The experiments were repeated twice.

### Extraction of total RNA, reverse transcription and quantitative real time RT-PCR

Fully-expanded upper leaves were collected at 2, 4, 6, 8, 10, 12, 14, 16 dpi with TCV, after systemic infection was successfully established with visual symptoms. Total RNAs were extracted using TRIzol reagent (Invitrogen) and ~3 μg was used to generate cDNAs through reverse transcription, using oligo(dT)_15_ or gene specific reverse primers with SuperScript^®^ III Reverse Transcriptase kit (Invitrogen^TM^). TCV CP fragment was amplified with appropriate primers listed in Table 2. Expression levels of the selected transcripts were analyzed via qRT-PCR. The qRT-PCR was set up in a total volume of 5 μl in a 384-well plate and carried out on the CX384^TM^ Real-Time PCR Detection System. Each reaction consisted of 1 μl of cDNA template, 0.1 μl each of both gene specific forward and reverse primers (10 μM) (Table 2), and topped up to 5 μl with water. CBP20 and Tubulin genes were used as internal controls for all qRT-PCR (Table 2). Each test consisted of three biological sample repeats and each sample contained three replicates.

### *Agrobacterium tumefaciens*-mediated transient expression and extraction of total proteins

*Agrobacteria* culture grown to OD_600nm_ = 1.0–1.5 was harvested, containing pGreen-GFP and pGreen-WRKY61-GFP. The cell pellet was resuspended in buffer (pH = 7) containing 10 mM each of MgCl_2_ and MES, and 100 μM acetosyringone, infiltration was performed after 4 h. Plasmid 35S pGreen + GFP was used as a negative control. All the infiltration transient expression experiments were repeated twice. *Arabidopsis* leaves were homogenized in the ratio of 0.1 g of leaf to 0.2 ml of protein extraction buffer [(220 mM Tris–HCl, pH 7.4, 250 mM sucrose, 50 mM KCl, 1 mM MgCl_2_, 2 mM phenylmethylsulfonyl fluoride, 10 mM β-mercaptoethanol, and 1 × complete EDTA-free protease inhibitor. The homogenate was centrifuged for 10 min, 4 °C, at 16.1 × 10^3^ *g*. The supernatant was separated to use directly or store at −20 °C.

### Western blot analysis of TCV CP

Western blot was carried out according to previously published protocol^64^. Briefly, newly emerged leaves from the inoculated plants at 10 dpi were collected for protein extraction. The denatured protein samples were separated on the 12% SDS PAGE gel and transferred onto a nitrocellulose membrane, followed by incubation with primary antibody anti-TCV antibody and secondary goat anti-rabbit antibody linked with alkaline phosphatase-conjugated protein A, and finally visualized using nitroblue tetrazolium/5-bromo-4-chloroindol-3-yl phosphate.

## Additional Information

**Data Availability**: Raw sequencing data is available through the NCBI Sequence Read Archive (BioSample Submission Portal, accession number: SAMN03516187). All samples were sequenced as 101 bp paired-end reads on an Illumina HiSeq2000 sequencer.

**How to cite this article**: Gao, R. *et al.* Genome-wide transcriptomic analysis reveals correlation between higher WRKY61 expression and reduced symptom severity in Turnip crinkle virus infected *Arabidopsis thaliana*. *Sci. Rep.* **6**, 24604; doi: 10.1038/srep24604 (2016).

## Supplementary Material

Supplementary Information

Supplementary Table S1

Supplementary Table S2

Supplementary Table S3
