# Author Correction: Genome-wide transcriptomic analysis reveals correlation between higher WRKY61 expression and reduced symptom severity in Turnip crinkle virus infected *Arabidopsis thaliana*

**DOI:** 10.1038/s41598-020-75427-5

**Published:** 2020-11-10

**Authors:** Ruimin Gao, Peng Liu, Yuhan Yong, Sek-Man Wong

**Affiliations:** 1 Department of Biological Sciences, National University of Singapore, Singapore, Singapore; 2 Temasek Life Sciences Laboratory, Singapore, Singapore; 3 National University of Singapore Suzhou Research Institute, Suzhou Industrial Park, Jiangsu, China

Correction to: *Scientific Reports* 10.1038/srep24604, published online 18 April 2016

This Article contains an error in Figure 4c, where the images of WRKY61-GFP for GFP and DAPI have been inadvertently switched. The correct Figure 4c appears here as Figure 1.

**Figure 1:**
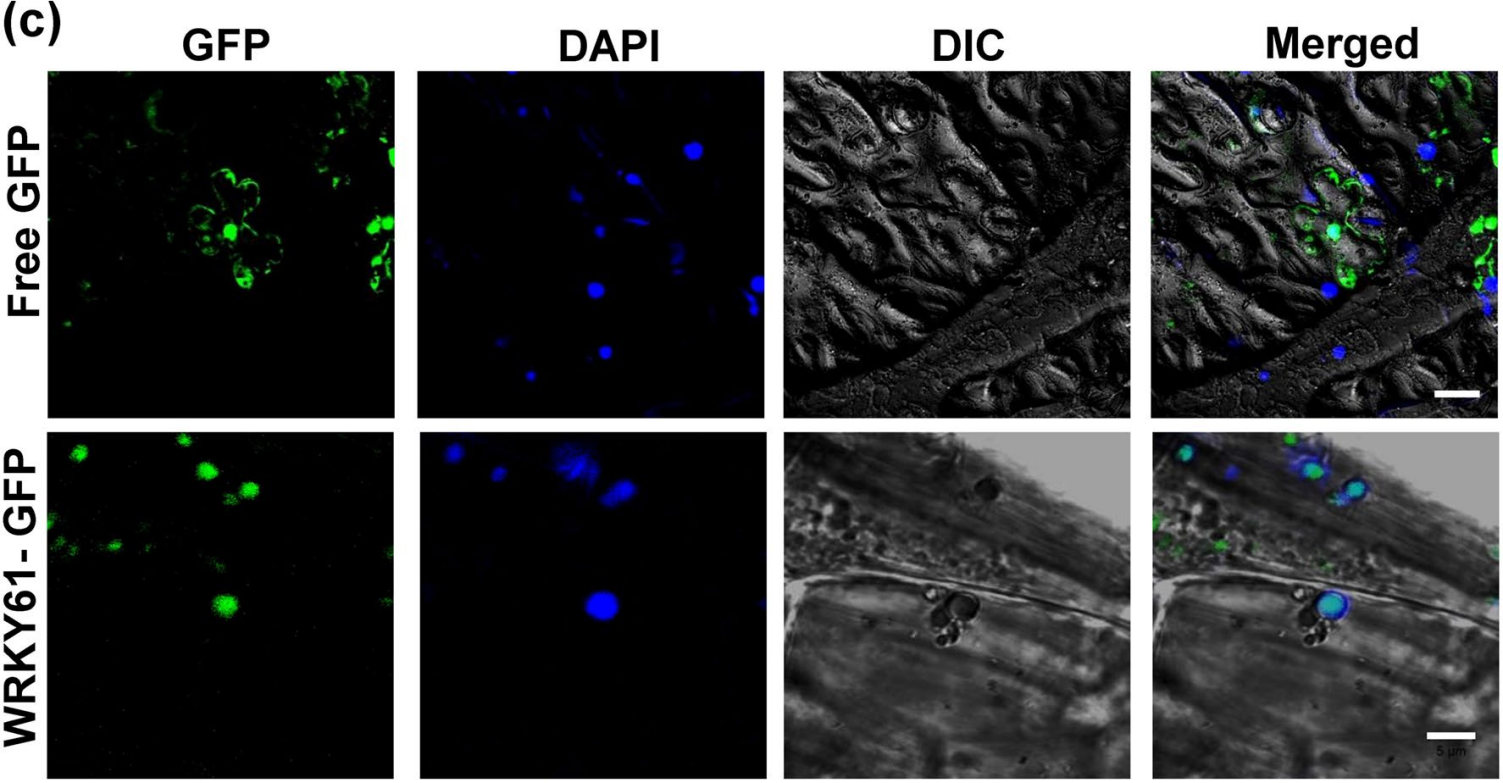
Relative gene expression level of WRKY61 in TCV-infected *Arabidopsis* and nuclear localization of WRKY61-GFP fusion protein in *Nicotiana benthamiana* leaves. (**c**) DAPI-stained nuclei (blue-color foci) were superimposed onto the differential interference contrast (DIC) image to form a merged image. *N. benthamiana* leaves were infiltrated with free GFP and the fluorescent signal was present in the entire cell including nucleus; WRKY61-GFP fusion proteins were only detected in the nucleus. Free GFP represents agro-infiltration with vector lacking of inserted gene. Bar = 5 μm.

