# Supplementary material for: Genome-wide transcriptomic analysis reveals correlation between higher WRKY61 expression and reduced symptom severity in Turnip crinkle virus infected Arabidopsis thaliana
Source: Sci Rep. 2016 Apr 18;6:24604. doi: 10.1038/srep24604 (PMC4834565; doi:10.1038/srep24604)
Supplement: Supplementary Information [file srep24604-s1.pdf]

Genome-wide transcriptomic analysis reveals correlation between higher WRKY61 expression and reduced symptom severity in Turnip crinkle virus infected *Arabidopsis thaliana*

Ruimin Gao, Peng Liu, Yuhan Yong, and Sek-Man Wong

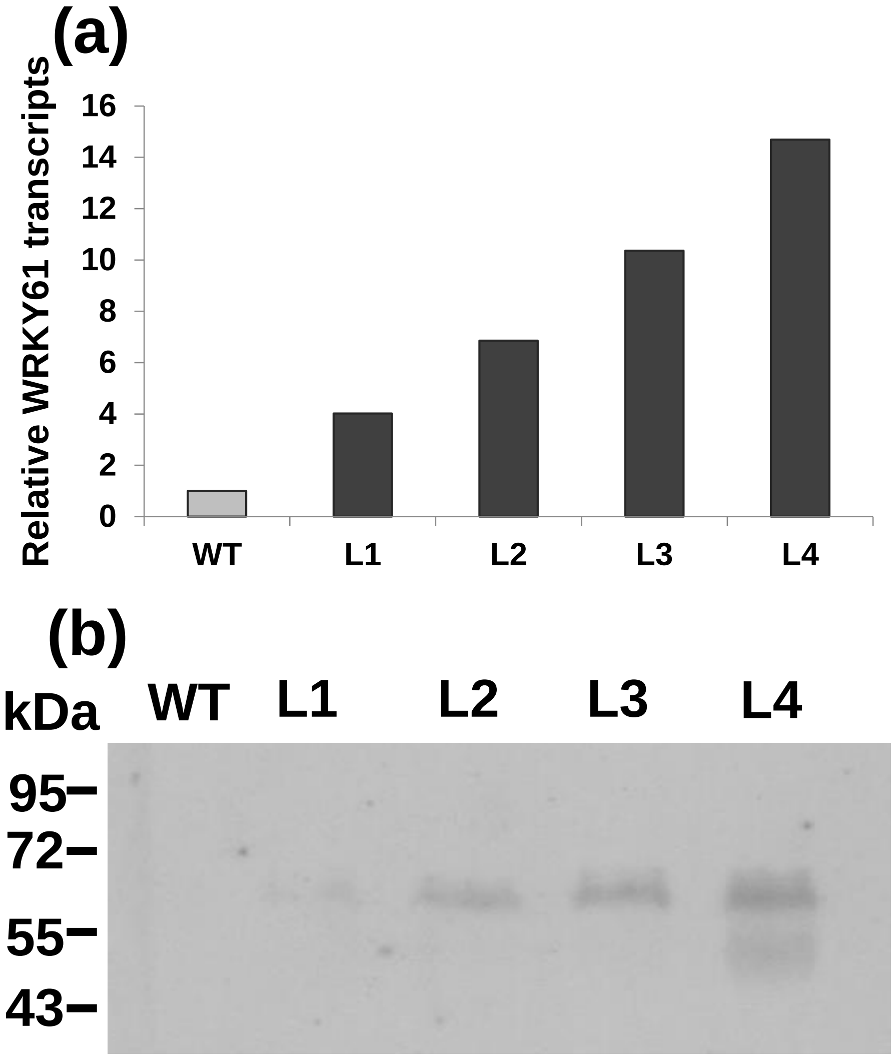

Supplementary Figure S1 Verification of WRKY61-6HA overexpression transgenic Arabidopsis. (a) Relative WRKY61 gene transcript levels (*CBP20* and *Tubulin* as internal controls) were analyzed using $2^{-\Delta\Delta C_T}$ method. (b) Detection of WRKY61-6HA fusion protein using anti-HA antibody. WT: wild type. L1-L4 represent different transgenic lines of Arabidopsis with WRKY61-6HA fusion protein.

Genome-wide transcriptomic analysis reveals correlation between higher WRKY61 expression and reduced symptom severity in Turnip crinkle virus infected *Arabidopsis thaliana*

Ruimin Gao, Peng Liu, Yuhan Yong, and Sek-Man Wong

Supplementary Document 1: RNA-seq data analysis materials

I: RNA-Seq raw reads QC results (per-base quality)

mock read 1 (Forward)

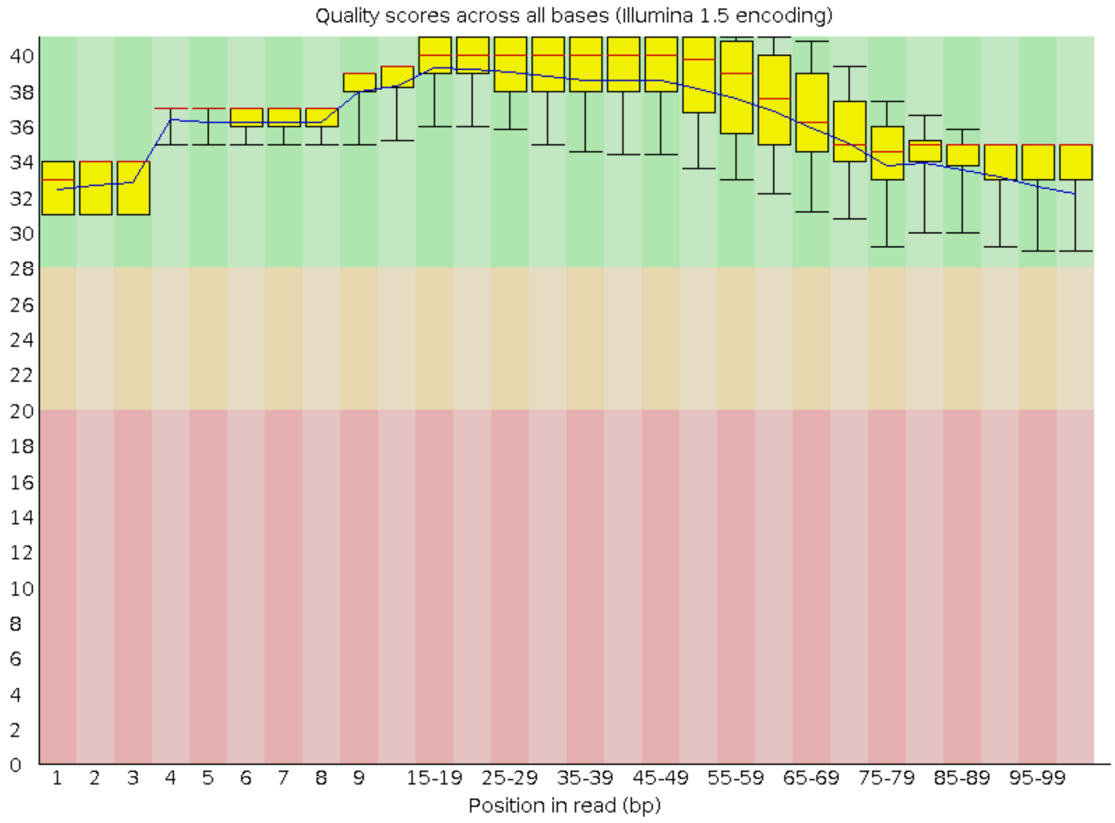

mock read 2 (Reverse)

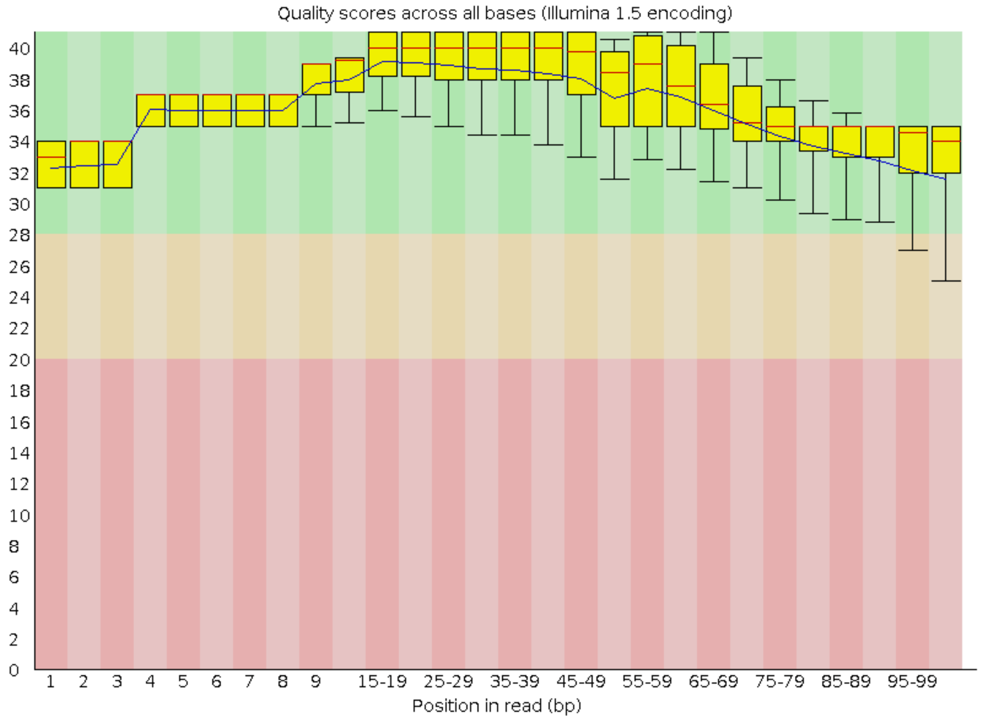

TCV reads 1 (Forward)

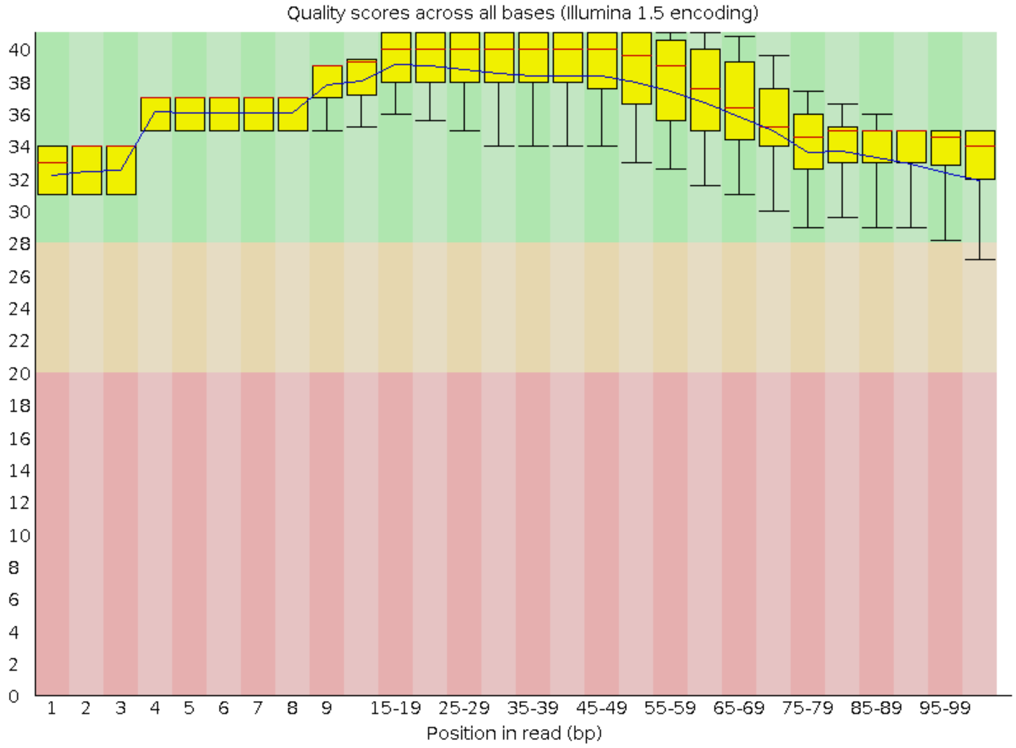

TCV reads 2 (Reverse)

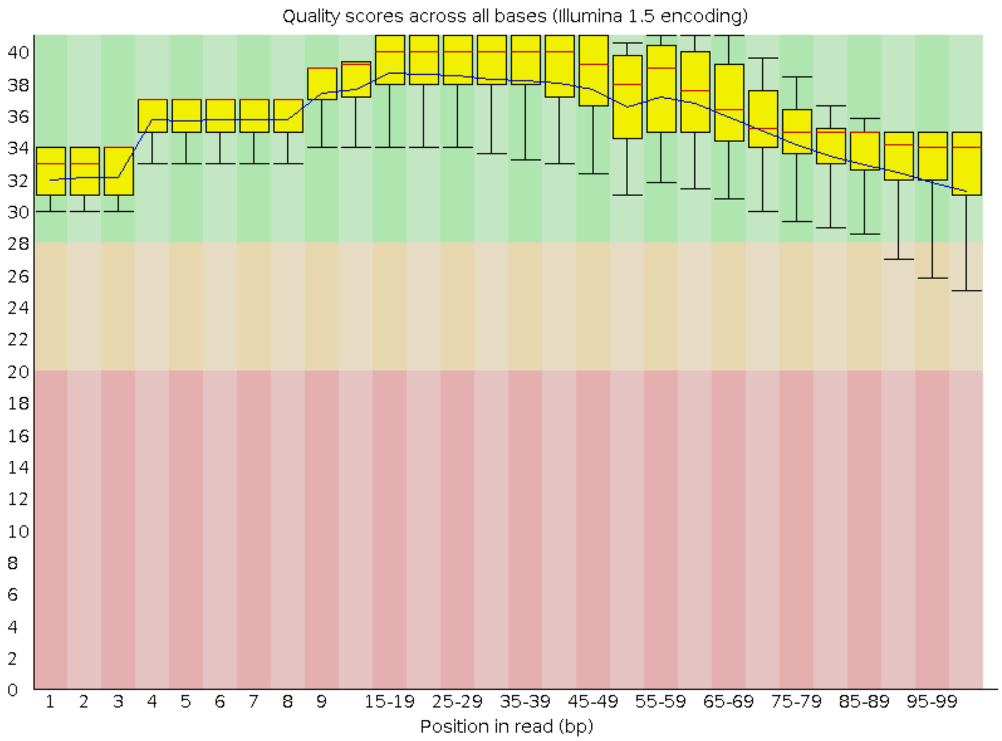

mock Read 1:

PASS Basic Statistics mock_1.fq.gz

PASS Per base sequence quality mock_1.fq.gz

PASS Per sequence quality scores mock_1.fq.gz

FAIL Per base sequence content mock_1.fq.gz

FAIL Per base GC content mock_1.fq.gz

PASS Per sequence GC content mock_1.fq.gz

PASS Per base N content mock_1.fq.gz

PASS Sequence Length Distribution mock_1.fq.gz

FAIL Sequence Duplication Levels mock_1.fq.gz

WARN Overrepresented sequences mock_1.fq.gz

WARN Kmer Content mock_1.fq.gz

mock read 2:

PASS Basic Statistics mock_2.fq.gz

PASS Per base sequence quality mock_2.fq.gz

PASS Per sequence quality scores mock_2.fq.gz

FAIL Per base sequence content mock_2.fq.gz

FAIL Per base GC content mock_2.fq.gz

PASS Per sequence GC content mock_2.fq.gz

PASS Per base N content mock_2.fq.gz

PASS Sequence Length Distribution mock_2.fq.gz

FAIL Sequence Duplication Levels mock_2.fq.gz

WARN Overrepresented sequences mock_2.fq.gz

WARN Kmer Content mock_2.fq.gz

TCV Read 1:

PASS Basic Statistics mock_2.fq.gz

PASS Per base sequence quality mock_2.fq.gz

PASS Per sequence quality scores mock_2.fq.gz

FAIL Per base sequence content mock_2.fq.gz

FAIL Per base GC content mock_2.fq.gz

PASS Per sequence GC content mock_2.fq.gz

PASS Per base N content mock_2.fq.gz

PASS Sequence Length Distribution mock_2.fq.gz

FAIL Sequence Duplication Levels mock_2.fq.gz

WARN Overrepresented sequences mock_2.fq.gz

WARN Kmer Content mock_2.fq.gz

TCV Read 2

PASS Basic Statistics virus_2.fq.gz

PASS Per base sequence quality virus_2.fq.gz

PASS Per sequence quality scores virus_2.fq.gz

FAIL Per base sequence content virus_2.fq.gz

FAIL Per base GC content virus_2.fq.gz

PASS Per sequence GC content virus_2.fq.gz

PASS Per base N content virus_2.fq.gz

PASS Sequence Length Distribution virus_2.fq.gz

FAIL Sequence Duplication Levels virus_2.fq.gz

WARN Overrepresented sequences virus_2.fq.gz

WARN Kmer Content virus_2.fq.gz

II: Tophat alignment summary

Mock

Left reads:

Input: 155260083

Mapped: 151390295 (97.5% of input)

of these: 20789836 (13.7%) have multiple alignments (331 have >20)

Right reads:

Input: 155260083

Mapped: 151356721 (97.5% of input)

of these: 20778336 (13.7%) have multiple alignments (373 have >20)

97.5% overall read alignment rate.

Aligned pairs: 149669813

of these: 17502335 (11.7%) have multiple alignments

and: 158470 (0.1%) are discordant alignments

96.3% concordant pair alignment rate.

TCV

Left reads:

Input: 170379319

Mapped: 152532447 (89.5% of input)

of these: 10760144 (7.1%) have multiple alignments (440 have >20)

Right reads:

Input: 170379319

Mapped: 152453823 (89.5% of input)

of these: 10752126 (7.1%) have multiple alignments (460 have >20)

89.5% overall read alignment rate.

Aligned pairs: 150493084

of these: 7517350 (5.0%) have multiple alignments

and: 302293 (0.2%) are discordant alignments

88.2% concordant pair alignment rate.

III: Methods and parameters for analysing RNA-Seq data using tophat and cufflinks:

1. Map the reads for each sample to the reference genome

tophat -p 8 -G all.gff3 -r 60 -o 1th R1_001.fastq R2_001.fastq

2. Assemble transcripts for each sample with reference gene structure

cufflinks -g ../all.gff3 -p 8 -o mock_cl mock_tophat/accepted_hits.bam

cufflinks -g ../all.gff3 -p 8 -o TCV_cl TCV_tophat/accepted_hits.bam

3. Run Cuffcompare on all assemblies to create a single merged transcriptome annotation:

cuffcompare -o TCV -s At.fa -r ../all.gff3 -R -C mock_cl/transcripts.gtf TCV_cl/transcripts.gtf

4. Run Cuffdiff by using the merged transcriptome assembly along with the BAM from Tophat for each replicate

cuffdiff -o diff1 -b Mt.fa -p 8 –L mock,TCV -u 1_cuffcompare/\1.combined.gtf \./mock_tophat/accepted_hits.bam \./TCV_tophat/accepted_hits.bam

Genome-wide transcriptomic analysis reveals correlation between higher WRKY61 expression and reduced symptom severity in Turnip crinkle virus infected *Arabidopsis thaliana*

Ruimin Gao, Peng Liu, Yuhan Yong, and Sek-Man Wong

Supplementary Table S4 Comparison of RNA-seq data and qRT-PCR

Gene ID	Gene symbol	Gene name	Fold change qRT-PCR (CBP20)	qRT-PCR (Tubulin)	RNA-Seq
AT2G19900	ATNADP-ME1	NADP-malic enzyme 1 [Source:TAIR_LOCUS;Acc: AT2G19900]	8.97	9.87	11.92
AT3G28510	Tri Hyd	P-loop containing nucleoside triphosphate hydrolases superfamily protein;	8.85	9.72	10.55
AT1G66700	PXMT1	S-adenosyl-L-methionine-dependent methyltransferases superfamily protein	2.73	3.67	9.17
AT1G52690	LEA7	Late embryogenesis abundant protein (LEA) family protein	9.30	10.16	9.10
AT2G29470	ATGSTU3	glutathione S-transferase tau 3	6.62	7.48	8.66
AT2G26020	PDF1.2B	plant defensin 1.2b	-10.52	-9.67	-10.84
AT5G44430	PDF1.2C	plant defensin 1.2C	-7.99	-7.12	-10.70
AT3G16670	Ole e 1	Pollen Ole e 1 allergen and extensin family protein	-9.37	-8.51	-10.21
AT5G20630	GLP3	germin 3. Encodes a germin-like protein	-9.03	-8.21	-9.26
AT2G10940	F15K19.1	Bifunctional inhibitor/lipid-transfer protein/seed storage 2S albumin superfamily protein	-8.08	-7.24	-8.37
AT3G22380	TIC	time for coffee	-0.10	0.77	0.01
AT3G61220	SDR1	NAD(P)-binding Rossmann-fold superfamily protein	-0.11	0.74	0.00
AT5G52240	MSBP1	membrane steroid binding protein 1	0.42	1.30	0.00
AT5G53300	UBC10	ubiquitin-conjugating enzyme 10	0.16	1.09	0.00
AT3G18820	ATRABG3F	Ras small GTPase, Rab type	-0.06	0.80	-0.01

Genome-wide transcriptomic analysis reveals correlation between higher WRKY61 expression and reduced symptom severity in Turnip crinkle virus infected *Arabidopsis thaliana*

Ruimin Gao, Peng Liu, Yuhan Yong, and Sek-Man Wong

Supplementary Table S5 Primers used in this study

Primers	Sequences (5' to 3')
AtWRKY61F	CCGGAATTCATGGATGAGGCTAAAGAGGAA
AtWRKY61R	CGCGGATCCAGGGCTCTTCTCAGCTTCATT
qAtWRKY61F	ACAAAGGCGAAGATGAAGACAG
qAtWRKY61R	CGAACCACTTGGAACCTCTGA
TCVqRT3454F	TCAAGAACAGAACTGGCTCAAC
TCVqRT3580R	AAATGACAATCGTGCTCCCAC
TCV3388F	ACCTATGGCCAAGGAGCCAATGATGCC
TCV 4053R	GGGCAGGCCCCCCCCCGCGCGA
qUP-ATNADP-ME1F	CATACCTTCCTCTTTCTTGGTGC
qUP-ATNADP-ME1R	TTGCGGCTTTCCTCTACCGA
qUP-Phos hydF	AGTTTCCATAGGCGACACCG
qUP-Phos hydR	GTTGCTCCACTTTCCTGACCT
qUP-PXMT1F	ACAATCCTCGTATCAGAGAGCA
qUP-PXMT1R	GCACAACCGAAATCCGCTAC
qUP-LEA7F	GCAGGTAAGGAGAAGACCAGT
qUP-LEA7R	GCGAAGCCCTAAAGTGTGCT
qUP-GSTU3F	TGGTTGTCAAGAGCCCGTTG
qUP-GSTU3R	GGACTTTGCGGTAGAATAGGATTG
qdon-PDF1.2bF	TCACCTTTATCTACGCTGCTCT
qdon-PDF1.2bR	TTCCGCAAACTCCTGACCA
qdon-PDF1.2cF	GTCTGCTACCATCATCACCTTC
qdon-PDF1.2cR	ATGTCCCACTTGGCTTCTCG
don-PollenE1F	GTGTGGAAACCTAAACAGAGTCG
qdon-PollenE1R	AATCGTTGAGGAGTGTGGGAA

qdon-GLP3F	TTACGCAGGCATCAATGGC
qdon-GLP3R	AATGACTACAAGGACCTCAGAAGC
qdon-BioinhiF	GACACGCTGAAGTTAGGTGC
qdon-BioinhiR	GCTGAAGAGCAACAGGGACA
qTICF	GAAGTTCCAACCCTGTTCAAGA
qTICR	TATTGTAGCGGCAGCGTGAG
qSDR1F	GGCGAAGAAACATCCCGAGT
qSDR1R	AGCCAACCTCACAGGACTTG
qMSBP1F	TGAGGAGAAAGACTTGACTTGGG
qMSBP1R	GGTTCAGAGACAGATGCGGT
qUBC10F	CACTGGCAGGCAACGATAAT
qUBC10R	TTGGGATGGAACACCTTGGT
qRABG3F F	GTGGTGCTGATTGCTGTGTT
qRABG3F R	ATCTGATGGACTCGCCTGGA
qCBP20-F	CAAGCTGCGCCAACGAATTATGG
qCBP20-R	TTGTCGTTTCCGATGGTAGTCTCCT
qTUB-F	ATCCGTGAAGAGTACCCAGAT
qTUB-R	AAGAACCATGCACTCATCAGC
Gabi-Kat-150-LB	ATAATAACGCTGCGGACATCTACATTTT
LP GK-618E08-021918	TGCTGCGACGAGGAGGAGGTTG
RP GK-618E08-021918	TGGGGATTGAATACAAGGCTTTGAGT
